# Supplementary material for: Machine Learning Accelerates Crystallization for Structure Determination
Source: Angew Chem Int Ed Engl. 2026 May 4;65(25):e1218503. doi: 10.1002/anie.1218503 (PMC13266958; doi:10.1002/anie.1218503)

checkCIF/PLATON report

Structure factors have been supplied for datablock(s) lcz048

THIS REPORT IS FOR GUIDANCE ONLY. IF USED AS PART OF A REVIEW PROCEDURE FOR PUBLICATION, IT SHOULD NOT REPLACE THE EXPERTISE OF AN EXPERIENCED CRYSTALLOGRAPHIC REFEREE.

No syntax errors found. CIF dictionary Interpreting this report

Datablock: lcz048

Bond precision:	C-C = 0.0071 A		Wavelength=1.54184
Cell:	a=24.4595(4)	b=12.9956(2)	c=25.5356(4)
	alpha=90	beta=117.487(2)	gamma=90
Temperature:	100 K		

	Calculated	Reported
Volume	7200.6(2)	7200.6(2)
Space group	P 21/c	P 1 21/c 1
Hall group	-P 2ybc	-P 2ybc
Moiety formula	C29 H15 Ag3 F18 N6 O3	C15 H3 Ag3 F18 N6, C14 H12 O3
Sum formula	C29 H15 Ag3 F18 N6 O3	C29 H15 Ag3 F18 N6 O3
Mr	1161.08	1161.08
Dx,g cm-3	2.142	2.142
Z	8	8
Mu (mm-1)	14.236	14.236
F000	4464.0	4464.0
F000'	4485.34	
h,k,lmax	31,16,32	30,15,32
Nref	15445	14788
Tmin,Tmax	0.082,0.241	0.462,1.000
Tmin'	0.006	

Correction method= # Reported T Limits: Tmin=0.462 Tmax=1.000
AbsCorr = MULTI-SCAN

```
Data completeness= 0.957          Theta(max)= 78.189

R(reflections)= 0.0414( 13073)    wR2(reflections)= 0.1193( 14788)

S = 1.085                 Npar= 1067
```

The following ALERTS were generated. Each ALERT has the format
test-name_ALERT_alert-type_alert-level.
Click on the hyperlinks for more details of the test.

Alert level C

```
PLAT042_ALERT_1_C Calc. and Reported MoietyFormula Strings  Differ     Please Check
              Calc: C29 H15 Ag3 F18 N6 O3
              Rep.: C15 H3 Ag3 F18 N6, C14 H12 O3
PLAT213_ALERT_2_C Atom F023            has ADP max/min Ratio .....         3.4 prolat
PLAT601_ALERT_2_C Unit-Cell Contains Solvent Accessible VOIDS .LE.          71 Ang**3
PLAT911_ALERT_3_C Missing FCF Refl Between Thmin & STh/L=    0.600          26 Report
                0  2  0,   0  8  0,  20  4  0,  26  0  0, -21  4  1,  -7  8  1,
               24  5  1, -24  0  2, -22  4  2,  -2  0  2,  -2  1  2,   0  0  2,
               17  4  2,  23  5  2,  25  0  2,  15  4  3,  24  0  4,  11  3  5,
               20  0 10,  -7  5 11, -16 12 16, -18 11 18, -14 10 22, -14 10 23,
              -13 10 23, -22  0 28,
```

Alert level G

```
PLAT007_ALERT_5_G Number of Unrefined Donor-H Atoms .............           2 Report
              H012  H013
PLAT083_ALERT_2_G SHELXL Second Parameter in WGHT  Unusually Large      17.53 Why ?
PLAT242_ALERT_2_G Low    'MainMol' Ueq as Compared to Neighbors of       C01X Check
PLAT242_ALERT_2_G Low    'MainMol' Ueq as Compared to Neighbors of       C02R Check
PLAT242_ALERT_2_G Low    'MainMol' Ueq as Compared to Neighbors of       C02V Check
PLAT242_ALERT_2_G Low    'MainMol' Ueq as Compared to Neighbors of       C035 Check
PLAT242_ALERT_2_G Low    'MainMol' Ueq as Compared to Neighbors of       C036 Check
PLAT242_ALERT_2_G Low    'MainMol' Ueq as Compared to Neighbors of       C038 Check
PLAT242_ALERT_2_G Low    'MainMol' Ueq as Compared to Neighbors of       C026 Check
PLAT242_ALERT_2_G Low    'MainMol' Ueq as Compared to Neighbors of       C02C Check
PLAT242_ALERT_2_G Low    'MainMol' Ueq as Compared to Neighbors of       C02T Check
PLAT242_ALERT_2_G Low    'MainMol' Ueq as Compared to Neighbors of       C02Y Check
PLAT242_ALERT_2_G Low    'MainMol' Ueq as Compared to Neighbors of       C030 Check
PLAT242_ALERT_2_G Low    'MainMol' Ueq as Compared to Neighbors of       C034 Check
PLAT434_ALERT_2_G Short Inter HL..HL Contact F009     ..F01E     .       2.80 Ang.
                                          -1+x,y,-1+z  =      1_454 Check
PLAT434_ALERT_2_G Short Inter HL..HL Contact F00F     ..F00S     .       2.73 Ang.
                                                x,y,z  =      1_555 Check
PLAT720_ALERT_4_G Number of Unusual/Non-Standard Labels .........         148 Note
              Ag01    Ag02    Ag03    Ag04    Ag05    Ag06    F007    F008
              F009    F00A    F00B    F00C    F00D    F00E    F00F    F00G
              F00H    F00I    F00J    F00K    F00L    F00M    F00N    O00O
              F00P    F00Q    F00R    F00S    F00T    F00U    F00V    F00W
              F00X    F00Y    N00Z    O010    N011    O012    H012    O013
              H013    O014    F015    F016    N017    N018    F019    F01A
              N01B    O01C    F01D    F01E    N01F    N01G    N01H    N01I
              N01J    F01K    N01L    N01M    F01N    C01O    H01O    C01P
```

```
              C01Q    C01R    H01R    C01S    C01T    C01U    C01V    C01W
              H01W    C01X    C01Y    H01Y    C01Z    C020    H020    C021
              C022    H022    F023    C024    H024    C025    H025    C026
              C027    C028    H028    C029    C02A    H02A    C02B    C02C
              C02D    C02E    C02F    H02F    C02G    C02H    C02I    H02I
              C02J    C02K    C02L    C02M    H02M    C02N    H02N    C02O
              H02O    C02P    C02Q    H02Q    C02R    C02S    C02T    C02U
              C02V    C02W    H02W    C02X    H02X    C02Y    C02Z    H02Z
              C030    C031    C032    H03E    H03F    H03G    C033    H03A
              H03B    H03C    C034    C035    C036    C037    H037    C038
              C039    H039    C03A    H03D
PLAT883_ALERT_1_G Absent Datum for _atom_sites_solution_primary ..     Please Do !
PLAT910_ALERT_3_G Missing FCF Reflection(s) Below Theta(Min)[Deg]=       3.47 Note
                1  0  0,
PLAT912_ALERT_4_G Missing # of FCF Reflections Above STh/L=  0.600        543 Note
PLAT941_ALERT_3_G Average HKL Measurement Multiplicity ..........         2.9 Low
PLAT969_ALERT_5_G The 'Henn et al.' R-Factor-gap value ..........       2.996 Note
              Predicted wR2: Based on SigI**2  3.98 or SHELX Weight 10.99
PLAT978_ALERT_2_G Number C-C Bonds with Positive Residual Density.          0 Info
```

```
   0 ALERT level A = Most likely a serious problem - resolve or explain
   0 ALERT level B = A potentially serious problem, consider carefully
   4 ALERT level C = Check. Ensure it is not caused by an omission or oversight
  23 ALERT level G = General information/check it is not something unexpected

   2 ALERT type 1 CIF construction/syntax error, inconsistent or missing data
  18 ALERT type 2 Indicator that the structure model may be wrong or deficient
   3 ALERT type 3 Indicator that the structure quality may be low
   2 ALERT type 4 Improvement, methodology, query or suggestion
   2 ALERT type 5 Informative message, check
```

Validation response form

Please find below a validation response form (VRF) that can be filled in and pasted into your CIF.

```
# start Validation Reply Form
_vrf_PLAT042_lcz048
;
PROBLEM: Calc. and Reported MoietyFormula Strings  Differ     Please Check
RESPONSE: ...
;
_vrf_PLAT213_lcz048
;
PROBLEM: Atom F023            has ADP max/min Ratio .....        3.4 prolat
RESPONSE: ...
;
_vrf_PLAT601_lcz048
;
PROBLEM: Unit-Cell Contains Solvent Accessible VOIDS .LE.         71 Ang**3
RESPONSE: ...
;
_vrf_PLAT911_lcz048
;
```

```
PROBLEM: Missing FCF Refl Between Thmin & STh/L=    0.600         26 Report
RESPONSE: ...
;
# end Validation Reply Form
```

It is advisable to attempt to resolve as many as possible of the alerts in all categories. Often the minor alerts point to easily fixed oversights, errors and omissions in your CIF or refinement strategy, so attention to these fine details can be worthwhile. It is up to the individual to critically assess their own results and, if necessary, seek expert advice.

PLATON version of 17/09/2025; check.def file version of 17/09/2025

---

duplicate check

No duplication found

Datablock lcz048 - ellipsoid plot

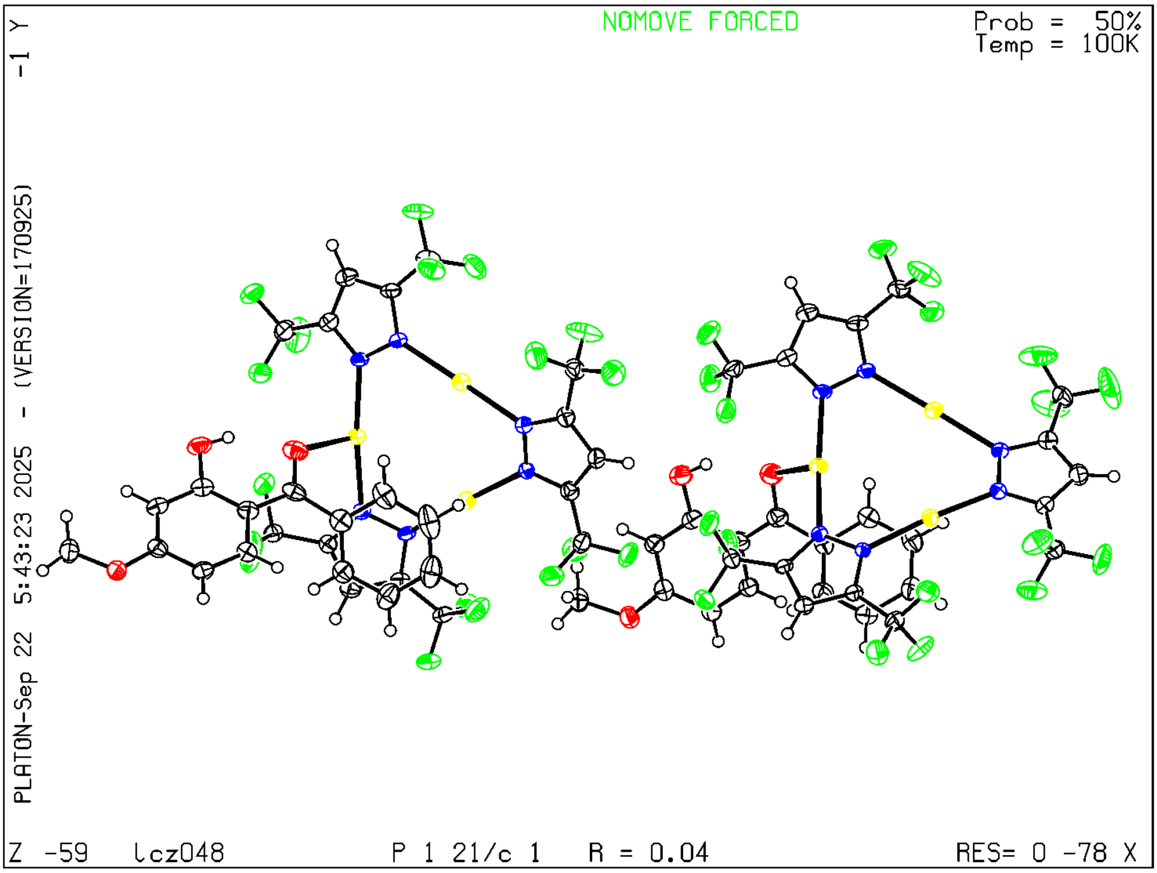

Supplement: Supplementary file 2 — Supporting File 2: anie72462‐sup‐0002‐DataFile.zip. [file ANIE-65-e1218503-s002.zip › supplementary_materials_crystallographic_data submit.zip!/cif_and_checkcif(1-114)/Ag3Pz3_48/Ag3Pz3_48_checkcif.pdf]
